# Genetic testing in adults with developmental and epileptic encephalopathy – what do we know?

**DOI:** 10.1515/medgen-2022-2144

**Published:** 2022-09-22

**Authors:** Ilona Krey, Kathrine M. Johannesen, Oona Kohnen, Johannes R. Lemke

**Affiliations:** Institute of Human Genetics, University of Leipzig Medical Center, Philipp-Rosenthal-Straße 55, 04103 Leipzig, Germany; Department of Epilepsy Genetics and Personalized Medicine, The Danish Epilepsy Centre, Dianalund, Denmark; Department of Regional Health Research, University of Southern Denmark, Odense, Denmark; Swiss Epilepsy Center, Klinik Lengg, Zurich, Switzerland; Center for Rare Diseases, University of Leipzig Medical Center, Leipzig, Germany

**Keywords:** adults, developmental and epileptic encephalopathy, epilepsy, genetic testing, NGS, precision medicine

## Abstract

Knowledge of underlying genetic causes of developmental and epileptic encephalopathies (DEE) in adults is still limited when compared to the routine diagnostic approach in similarly affected children.

A well-documented longitudinal study of adults with DEE is of utmost importance to understand the natural history of the respective entity. This information is of great value especially for genetic counselling of newly diagnosed children with identical genetic diagnoses and may impact treatment and management of affected individuals.

In our meta-analysis we provide an overview of the most recurrent genetic findings across an adult DEE cohort (n=1,020). The gene mostly associated with a pathogenic or likely pathogenic variant in adult DEE is *SCN1A*, followed by *MECP2* and *CHD2*. Studies employing exome sequencing and calling of both single nucleotide variants and copy number variants are associated with diagnostic yields of almost 50 %.

Finally, we highlight three remarkable cases, each representing the oldest individual ever published with their genetic diagnosis, i. e., Angelman syndrome, Miller–Dieker syndrome, and *CAMK2A*-related disorder, and describe lessons learned from each of these adults.

## Introduction

Developmental and epileptic encephalopathies (DEE) are severe disorders with typically early-onset epileptic seizures and intellectual disability (ID) [1].

The prevalence of epilepsy in individuals with ID is estimated to be 22 %, increasing with the degree of ID [2].

Within recent years, genetic testing has become part of the routine diagnostic approach in children with DEE [3], resulting in a huge and growing number of published pediatric cohorts and an enormous improvement in our knowledge of underlying genetic disorders of DEE in young individuals.

In comparison, adults with DEE are rarely tested genetically and published studies about their genotypic or phenotypic spectrum are scarce. Even though knowledge is limited, it is increasingly recognized that genetic testing is of great importance for adults with DEE, as seen by a slight increase in recent publications focusing on genetic causes of DEE in adults and even elderly.

Among other important facts, recent studies described that genetic testing, especially next-generation sequencing (NGS), reduces time and costs associated with the diagnostic journey [4], [5], a fact that has received less attention in adult care.

The aim of this study was to summarize existing and unpublished knowledge of genetic testing in adults with DEE, to identify recurrent genetic findings within this cohort, and to highlight some remarkable or unusual cases.

## Methods

### Previously published cases

A PubMed search on “adults, genetic testing and epilepsy” on 14 April 2022 delivered more than 400 entries. Papers not available in English were excluded. Only four studies described the genotypic spectrum of DEE in adult individuals [6], [7], [8], [9]. Only original cases with pathogenic or likely pathogenic [10], [11] single nucleotide variants (SNVs) and/or copy number variants (CNVs) according to the guidelines of the American College of Medical Genetics and Genomics and the Association for Molecular Pathology (ACMG-AMP) [10], [11] were included.

### Additional novel cases

An in-house cohort of 232 adult individuals with epilepsy and ID was merged with the data of the previously mentioned four publications [6], [7], [8], [9]. Individuals were recruited through routine diagnostic exome sequencing at the Institute of Human Genetics at the University of Leipzig, Germany. Genetic testing comprised single exome sequencing with the Human Core Exome hybridization probes from Twist Bioscience. Sequencing was performed on a NovaSeq 6000 Instrument using an S1 Reagent Kit (300 cycles) by Illumina. Analysis of the raw data was performed using the software Varfeed (Limbus, Rostock). Analysis included SNV and CNV analysis. Detected SNVs and CNVs were evaluated according to the guidelines of the ACMG-AMP and ClinGen [10], [11]. For all cases legal guardians gave their written informed consent. The study was approved by the ethics committee of the University of Leipzig and the Cantonal Ethics Committee of Zurich (224/16-ek, 402/16-ek, KEK-ZH-Nr. 2011-0482).

## Results

When reviewing the literature on studies on genetic testing in adults with DEE, only four relevant publications were found [6], [7], [8], [9]. Table 1 shows the respective diagnostic yield and the applied testing methods. All four studies were combined with our unpublished cohort, yielding a total of 1,020 adults and elderly with DEE. This cohort size enables detection of some recurrent genetic findings in adult individuals with DEE.

For 294 individuals a pathogenic or likely pathogenic variant resulting in a genetic DEE diagnosis could be identified, providing an overall diagnostic yield of 28.8 %.

**Table 1:** Diagnostic yield and testing methods of the four published studies and our unpublished cohort of genetic testing in adults with DEE.

Cohort size	Solved cases	Diagnostic yield in %	Testing method	Reference
438*	70	**16.0**	Gene panel (only SNVs) (89–133 genes)	McKnight et al., 2022 [9]
150	71 (49 SNVs, 23 CNVs)	**47.3**	CA, *FMR1*; CMA, gene panel; ES for all unresolved cases (n=93)	Zacher et al., 2021 [8]
200	46	**23.0**	Gene panel (only SNVs) (45–580 genes)	Johannesen et al., 2020 [7]
64*	14	**21.9**	Gene panel (only SNVs) (126–185 genes)	Borlot et al., 2019 [6]
232	107 (92 SNVs, 15 CNVs)	**46.1**	Exome sequencing (SNV and CNV analysis)	In-house cases from routine diagnostics

CA = conventional karyotyping, CMA = chromosomal microarray, ES = exome sequencing.

*All 64 individuals from Borlot et al. (2019) were also part of the 438 individuals of McKnight et al. (2022).

The diagnostic approaches differ in the reviewed studies: In three of four studies [6], [7], [9] panel analysis was the applied testing method with the number of included genes ranging from 45 to more than 500 for SNV analysis. Of note, CNV analysis was not part of these studies. Only two sub-cohorts investigated both SNVs and CNVs. In the respective studies (likely) pathogenic CNVs were found in 15.3 % (n=23 of 150 [8]) and 6.7 % (n=15 of 232, in-house data). Both these studies had a remarkably higher yield of (collectively) 46.6 % solved cases [8] compared to a collective diagnostic yield of 18.2 % of studies solely analyzing SNVs [6], [7], [9].

The gene mostly associated with a pathogenic or likely pathogenic variant in this cohort of 1,020 DEE cases was *SCN1A* (n=62), followed by *MECP2*, *CHD2*, *DEPDC5*, *UBE3A*, *PRRT2*, *PCDH19*, *STXBP1*, and numerous other genes, each with less than eight cases (corresponding to a prevalence of <0.7 %) within the overall cohort (Figure 1). Thus, *SCN1A* remains the by far most relevant gene also in adults with genetically determined epilepsy disorders, in particular DEE. Only few diagnoses are highly recurrent, whereas the vast majority comprises rare or even unique diagnoses.

**Figure 1:** Distribution of recurrent genetic findings across genes within the overall adult DEE cohort (n=1,020).

Among those with a diagnostic finding, frequency was calculated by gene. The number of individuals with a diagnostic finding in a gene is indicated in parentheses along the *x*-axis. Genes found in only one individual are not shown in the table.

Whether called “disease-associated treatment approaches,” “gene-specific treatment changes,” “precision medicine,” or “clinically actionable findings,” in up to 50 % of individuals who received a genetic diagnosis, treatment approaches and recommendations (such as change in medication, screening for disease-associated malformations, cardiac arrhythmia, and/or potential tumor predispositions, etc.) changed due to the newly diagnosed underlying genetic disease [6], [7], [8], [9]. In pediatric cohorts, the fraction of cases where treatment is changed as a consequence of a genetic diagnosis can reach 80 % [12].

A diagnostic finding that had clinical management implications beyond epilepsy care (secondary findings) was described in 3–4 % of individuals, for example a (likely) pathogenic variant in *BRCA2* which is associated with a higher risk for breast and ovarian cancer [8], [9]. Another example was a pathogenic variant in *KCNH2* which is associated with long QT syndrome and sudden cardiac death [13], leading to cardiologic monitoring.

## Remarkable cases

### Case 1 – Angelman syndrome

Individual #1 is an 84-year-old female born in 1937 with moderate ID living in a nursing home since her mother died several years ago. In early childhood during the late 1930s and 1940s in Germany, she was kept hidden by her family in order to protect her. Only marginal information is available on her early life. She had several events interpreted as seizures in childhood that never re-occurred in adulthood. Anti-seizure medication was not initiated. At the age of 5 years, she was able to walk independently. She was never able to speak. Genetic testing at the age of 77 years revealed a paternal uniparental disomy 15 and thus the diagnosis of Angelman syndrome. After decades of stability of her health conditions, her motor skills are still mildly impaired due to a COVID-19 infection last year. Since this time, depending on her state of health she mostly depends on a wheelchair and is incontinent. She understands and follows simple instructions, is able to eat and drink by herself, and communicates by gestures, e. g., pointing with her finger.

To the best of our knowledge, she is the oldest individual ever reported with Angelman syndrome. She was already a 27-year-old adult when Angelman syndrome was first described [14] in 1965. Her records over many decades thereafter revealed a stable and non-progressive course of Angelman syndrome into very old age.

### Case 2 – No longer Rett syndrome

Individual #2 is a 43-year-old male with severe ID, epilepsy with complex focal seizures, spastic tetraparesis, scoliosis, and retinitis pigmentosa. As part of a research project a rare variant in *MECP2* was detected in 2012, leading to suspicion of Rett syndrome. Despite the absence of any diagnostic verification, this suspicion was adopted throughout his medical records. Because of doubts on the diagnosis of Rett syndrome, he was re-sequenced in 2021. Exome sequencing revealed a known [15] pathogenic gain-of-function missense variant (NM_015981.3:c.845A>G, p.(His282Arg)) in *CAMK2A* [15], changing the diagnosis into mental retardation, autosomal dominant 53 (OMIM #617798), first described in 2017. Up to now only 14 unrelated individuals with pathogenic variants in *CAMK2A* and neurodevelopmental disorders with or without epilepsy have been reported. As none of these 14 individuals is adult, our case represents the oldest individual with this *CAMK2A*-related disorder showing ongoing seizures also into adulthood as part of the phenotypic spectrum. We also detected a rare variant in *MECP2* (NM_004992.3: c.1358G>A, p.(Arg453Gln)) and assumed that this is the variant found in 2012. This variant is reported twice in the general population, once in a healthy male (gnomAD). Because of the severe phenotype of disease-causing variants in *MECP2*, we conclude that this variant is likely benign. The diagnosis of *MECP2*-associated Rett syndrome was therefore corrected and updated to *CAMK2A*-related disorder. At the time of the genetic diagnosis in 2012, *CAMK2A* was not yet described as a disease gene. Thus, this case illustrates the importance of re-interpretation or even re-analysis of unclear cases, preferably by exome sequencing.

### Case 3 – Miller–Dieker syndrome

Individual #3 is a 46-year-old male with severe ID and onset of intractable unclassified epilepsy with epileptic spasms, tonic, myoclonic, and bilateral tonic-clonic seizures with focal onset at 6 months of age. At the time of initiation of genetic testing, he had between 10 and 30 seizures per month despite treatment with valproic acid (500 mg/day), lamotrigine (125 mg/day), and perampanel (6 mg/day, initiated in 2015). Computed tomography in early childhood revealed lissencephaly and hydrocephalus internus. Additional diagnoses include spastic tetraplegia, scoliosis, and sleep apnea, and he has dysmorphic facial features (tall prominent forehead, bitemporal narrowing, hypertelorism, short nose, broad and thick upper lip). He lives at a residence for adults with ID and epilepsy. His parents describe him as a very happy and content person, he often smiles and likes to drive around with his wheelchair. He is not able to walk or sit alone and he communicates non-verbally. Genetic testing revealed a *de novo* 277-kb heterozygous pathogenic deletion in 17p13.3 including *PAFAH1B1*. Although *YWHAE* was not deleted, the clinical presentation led to diagnosis of Miller–Dieker lissencephaly syndrome (MDS). The currently oldest known individual with MDS died at age 17 years. In isolated *PAFAH1B1*-associated lissencephaly, approximately 50 % live to the age of 10 years, and very few reach the age of 20 years. The currently oldest known individual with *PAFAH1B1*-associated lissencephaly lived to the age of 30 years. Thus, individual #3 is the oldest case ever described with this disorder. Ikemoto et al. [16] described five individuals with *PAFAH1B1*-associated lissencephaly, including two subjects with MDS, and a seizure reduction of ≥50 % was reported in four of five patients after treatment with perampanel. Myoclonic seizures even disappeared in one individual. They proposed perampanel as an effective adjunctive anti-seizure medication. Individual #3 already received perampanel in 2015 leading to a slight decrease in seizure frequency from >30 seizures per month down to 10–30 seizures per month, being in line with the later observation by Ikemoto et al. [16].

## Discussion and conclusion

There is a need to increase awareness of access to genetic testing among adults with DEE, regardless of age. The results of the abovementioned studies demonstrate that genetic testing in adults with DEE is worth undertaking.

Most adults with DEE have been through a long and unfinished diagnostic odyssey, where caregivers and neurologists have given up on finding a definite diagnosis. Often the clinical history is well documented for decades.

Thus, these individuals often live unnoticed, e. g., in a residence, and genetic testing is often not considered an option. However, as the above findings show us, there are several aspects (definite diagnosis, impact of treatment and management of affected individuals and newly diagnosed children, etc.) of genetic testing in adults with DEE that make it worthwhile.

In Zacher et al. [8], all CNVs detected by chromosomal microarray were also detected via CNV analysis of NGS data. Additional causative variants in unsolved cases sequenced with panel analysis could be identified in approximately 10 % (n=13) by exome sequencing, underlining the need to rethink the importance of several genetic testing methods [8].

Therefore, exome sequencing should be the first diagnostic step [5], [17], and conventional genetic testing methods (such as analysis of repeat expansions, UPD testing, etc.) can be considered as a second step in unsolved cases. NGS-based sequencing techniques, especially exome sequencing, should be considered as first-tier diagnostic approaches in DEE, as justified by the high diagnostic yield discussed in Krey et al. [18].

Even though case numbers are still relatively low, analysis with smaller gene panels turned out to result in a lower diagnostic yield compared to larger gene panels or even exomes. Still, the total diagnostic yield of 28.8 % in DEE is likely an underestimate, which is illustrated by a marked increase of diagnostic yield up to almost 50 % when both SNVs and CNVs are investigated. Additionally, the panel design influences the outcome, especially in small numbers of analyzed genes. It is also possible that many monogenic cases of DEE have a severe course and do not reach adulthood, while entities that are not monogenic may have a milder disease course (see Krey et al. [19]) and are more likely to grow old.

A higher diagnostic yield in the respective cohorts was seen in females [9], in individuals with severe ID, and in individuals with anecdotal evidence of exogenic early-life events (e. g., nuchal cord, complications at delivery) [8]. McKnight et al. [9] cite disease-causing variants in X-linked genes (such as *MECP2* and *PCDH19*) affecting females at a greater rate than males and a possibly elevated mortality rate of affected male fetuses. A similar diagnostic yield was observed in individuals with childhood- and adult-onset seizures, both of whom had a higher diagnostic yield than those with adolescent-onset seizures [9].

Misdiagnosis, especially in case of alleged vaccine encephalopathy, is more than relevant in perspective of the recent pandemic. The fear that vaccination may cause encephalopathies is well known. However, this hypothesis was widely refuted by Berkovic et al. [20], showing that 11 of 14 individuals (78.6 %) with alleged vaccine encephalopathy in fact had Dravet syndrome due to a pathogenic variant in *SCN1A* [6]. Moreover, individuals with DEE due to unproven exogenic factors (e. g., teratogenic medications, maternal infection during pregnancy, nuchal cord, complications at delivery, central nervous system trauma, or vaccination) and normal MRI were found to have an increased diagnostic yield of 58.3 % of monogenic diagnoses, pointing towards a positive predictive value for a genetic etiology in this subset of individuals [8].

Therefore, the proportion of undiagnosed adults with DEE and cases without access to genetic testing is probably even larger than assumed, which is why the indication for genetic testing should be reconsidered by clinicians who see and treat adults with DEE.

Although therapeutic options for DEE are still limited [21], the rapid progress that has been made in recent years in this field, additionally to the importance of improved quality of life in every single successfully diagnosed and treated case, allows enthusiasm in expanding the knowledge and approaches in the near future.

A frequent example of precision medicine approaches is the treatment of patients with Dravet syndrome due to loss-of-function variants in *SCN1A*, which often has a typical presentation in childhood but can be much less distinguishable from other DEE in adults. Still, avoiding sodium channel blockers remains beneficial in adulthood, as illustrated in a 41-year-old female showing improvements in seizure control and language skills after a change of medication [7]. Another example is a 28-year-old individual carrying a pathogenic variant in *SLC2A1* (leading to GLUT1 deficiency) who improved significantly and even achieved seizure freedom after initiating ketogenic diet [7].

Besides Dravet syndrome, *KCNQ2*-related DEE is another challenging disorder due to changing clinical presentations from childhood to adulthood. The rate of diagnosed adults with pathogenic variants in *KCNQ2* is surprisingly low given that this disorder constitutes one of the most common genetic findings in pediatric cohorts. Johannesen et al. [7] speculated that this might be due to inclusion bias as adults with *KCNQ2*-related DEE frequently achieve seizure freedom and are thus less likely to be included in adult epilepsy cohorts. This observation was recently supported by a detailed phenotypic description of 13 adults with *KCNQ2*-related DEE [22].

In this respect, we describe three individuals of our overall cohort representing the oldest cases ever published with their individual diagnosis, i. e., Angelman syndrome, Miller–Dieker syndrome, and *CAMK2A*-related disorder. For Miller–Dieker syndrome and *CAMK2A*-related disorder no description of adults is known at all so far.

Another very important aspect is the relevance of diagnostic findings with clinical management implications beyond epilepsy care, also called “secondary findings” or “additional findings.” In the total cohort of 1,020 individuals this might be relevant for more than 30 individuals. Regarding the cognitive and physical deficits and often the inability of realizing and verbalizing relevant disease-associated symptoms this aspect is of special importance in adults with DEE.

Regardless of the age of the individuals with DEE, the ongoing rapid technological progress in genetic testing and the development of precision medicine approaches will be a driving force in the future. Genetic diagnostics, mainly exome or genome sequencing, for adults should become part of routine diagnostics, just as it is already established for the majority of pediatric DEE patients.

Knowledge of the underlying course will not only end a perhaps long-lasting diagnostic odyssey. Analysis of the disease course of adult or even elderly individuals with DEE allows for a better prediction of the outcome of newly diagnosed and usually very young individuals. Therefore, information on the disease course of DEE is of particular altruistic value [8].
